# A New Mirroring Circuit for Power MOS Current Sensing Highly Immune to EMI

**DOI:** 10.3390/s130201856

**Published:** 2013-01-31

**Authors:** Orazio Aiello, Franco Fiori

**Affiliations:** Microelectronics EMC Group, Eln. Department, Politecnico di Torino, Corso Duca degli Abruzzi, 24, I-10129 Torino, Italy; E-Mail: franco.fiori@polito.it

**Keywords:** current sensor, CMOS integrated circuit, smart power, electromagnetic interference (EMI), electromagnetic compatibility (EMC), senseFET, miller effect

## Abstract

This paper deals with the monitoring of power transistor current subjected to radio-frequency interference. In particular, a new current sensor with no connection to the power transistor drain and with improved performance with respect to the existing current-sensing schemes is presented. The operation of the above mentioned current sensor is discussed referring to time-domain computer simulations. The susceptibility of the proposed circuit to radio-frequency interference is evaluated through time-domain computer simulations and the results are compared with those obtained for a conventional integrated current sensor.

## 1. Introduction

In the last decades, the development of CMOS technologies in terms of scale integration and component performance has made the integration of full electronic system into a single chip possible. Such System-on-Chips (SoCs) comprise complex high-speed digital blocks, analog circuits and power section [1,2]. The power-supply voltage of such integrated circuits has decreased over time, leading to lower noise margins. However, the level of disturbances collected by the wiring harnesses of the electronic systems from the surrounding environment has strongly increased because of the widespread use of wireless systems for radio/TV broadcasting and mobile communications. In this context, the need to understand the effect of radio frequency interference (RFI) on baseband integrated circuits has continued to grow and several studies have tried to set out design guidelines and new circuit topologies for making integrated circuits immune to RFI [3–8]. Such disturbances superimposed onto nominal signals are of particular concern in the design of input and output front-end integrated circuits like those included in smart power ICs, which cannot be protected using EMI-filters at the PCB level because either their presence can affect the nominal circuit operation or the PCB is not present since the SoC is encapsulated inside the sensor (actuator) plastic holder. For instance, EMI filters and capacitors cannot be connected to the power transistor terminals because they would reduce the power efficiency. As a result, the power transistors that drive actuators through cables can experience high-level RFI that can lead to unexpected transistor switching and leakage currents, as shown in [9,10]. In addition to this problem, the circuits that are connected to the transistors' terminals, including circuits embedded in smart power ICs for monitoring the operation of the power transistors and the actuators, are also affected by RFI.

Within the aforementioned context, this paper investigates the susceptibility of integrated current sensors that are used in power circuits for control and/or safety purposes to RFI. The current of a power transistor can be sensed by using one of the circuits presented in [11], but only a few of them are suitable for integration on silicon. Among these integrated current sensors, one of the most frequently used is that based on a small transistor namely SenseFET. The voltages at the terminals of the SenseFET are kept equal to those of a power transistor in which the current to be detected flows. In this way, such a current is scaled by a constant *K* and provided by the SenseFET. *K* depends only on the aspect ratios $\left(\frac{W}{L}\right)$ of the two transistors. In this circuit, the SenseFET is electrically connected to the terminals of the power transistor and therefore it is affected by the RF disturbances collected by cables. In order to design embedded current sensor immune to such RF interferences, this paper presents a new integrated current-sensing solution conceived to be electrically not connected to the power transistor drain that can be affected by disturbances. The current is sensed whenever during its transient the power transistor and the SenseFET both operate in saturation. Therefore, the current flowing through the power transistor is mirrored by the SenseFET, is not dependent on the drain-source voltage and is only related with the mirroring factor K. The paper is organized as follows. Section 2 describes the operation of a common integrated current sensor. Section 3 summarizes the switching behavior of power MOSFETs. On the basis of such a behavior, a new current sensor not electrically connected to the drain terminal of the power transistor is proposed in Section 4. Then, the results of computer simulations aimed at evaluating the susceptibility of the proposed current sensor, the MagFET-based sensor and the commonly used current sensor to RFI are shown in Section 5. Finally, some concluding remarks are drawn in Section 6.

## 2. A Conventional Current Sensor: The SenseFET

**Figure 1.**
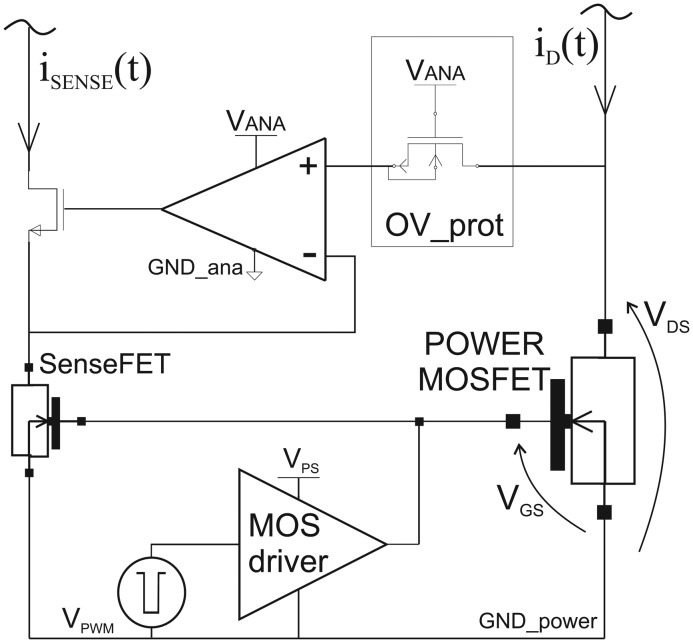
Current sensor based on the SenseFET technique.

Common current sensors for integrated applications are based on the SenseFET circuit topology, in which the current *i_D_* (*t*) that flows through an MOS Power transistor is sensed using an elementary transistor of the same type that is used in the power transistor. Figure 1 shows the operation of the SenseFET current sensor, where the gate terminals of the aforementioned transistors are connected to each other and driven by an MOS driver, while the drain-to-source voltage of the SenseFET is made equal to that of the power transistor by means of a voltage follower. In this circuit, the relationship between the power transistor current (*I*_D_) and the sensed current (*I*_SENSE_) can be written as

(1) $$K=\frac{{\left(\frac{W}{L}\right)}_{\text{power}}}{{\left(\frac{W}{L}\right)}_{\text{sense}}}$$

where ${\left(\frac{W}{L}\right)}_{\text{power}}$ and ${\left(\frac{W}{L}\right)}_{\text{sense}}$ are the aspect ratios of the power and SenseFET transistors, respectively. K is typically not lower than 10^3^. The scaled current *I*_SENSE_ is then converted to a voltage, using a resistor to be used for digital signal processing. This paper focuses on a low side sensing topology highlighted in Figure 1.

However, the same reasoning can be applied for the high side topologies. In this technique the power dissipation is low and the accuracy is related to the matching of the SenseFET and the Power MOS. The two transistors are driven in the deep triode region by the same gate-source voltage and have to be kept at the same drain-source voltages. Therefore, to minimize the error in the sensed current, the amplifier needs to have a very high gain. Moreover, the amplifier should maintain the accuracy across the large input common-mode range. Although several circuit topologies of current sensors have been proposed, all of them include an amplifier, which implies stability issues, limited bandwidth and increased design complexity. In fact, the improvements in integrated current-sensing are basically focused on increasing amplifier performance [12–19]. Furthermore, the presence of the amplifier increases the overall susceptibility of the commonly employed SenseFET-based current sensing to EMI. In fact, this amplifier is connected through an over-voltage protection (see Figure 1) to the power transistor drain, and hence to the wiring interconnects at the system level that collect EMI. Since interference with an amplitude of hundreds of mV causes distortion in the amplifier and in the over-voltage protection, the operation of the current sensor can be impaired [6].

For all the above mentioned reasons, a new current sensor with improved performance due to the absence of any amplifier has been designed and its immunity to EMI is specifically addressed in this paper.

## 3. Power MOSFET Switching Characteristics

**Figure 2.**
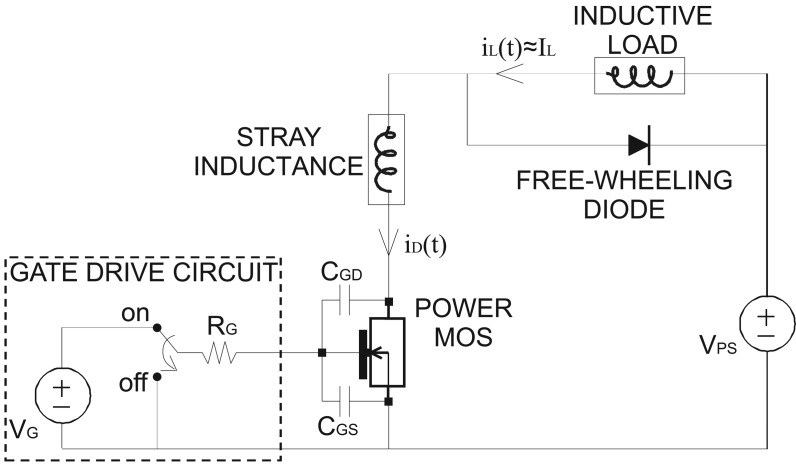
Power MOSFET device operating in an inductive load circuit.

The Power MOSFET is usually employed as a switch in circuits for energy conversion and management applications and to drive high-power loads. For instance, a Power MOSFET is used to control the current in inductive loads such as the windings of motors. A Power MOSFET that drives an inductive load is represented in Figure 2 where a freewheeling diode carries the load current when the Power MOS is switched-off and a stray inductance is included to account for package and board parasitic elements.

**Figure 3.**
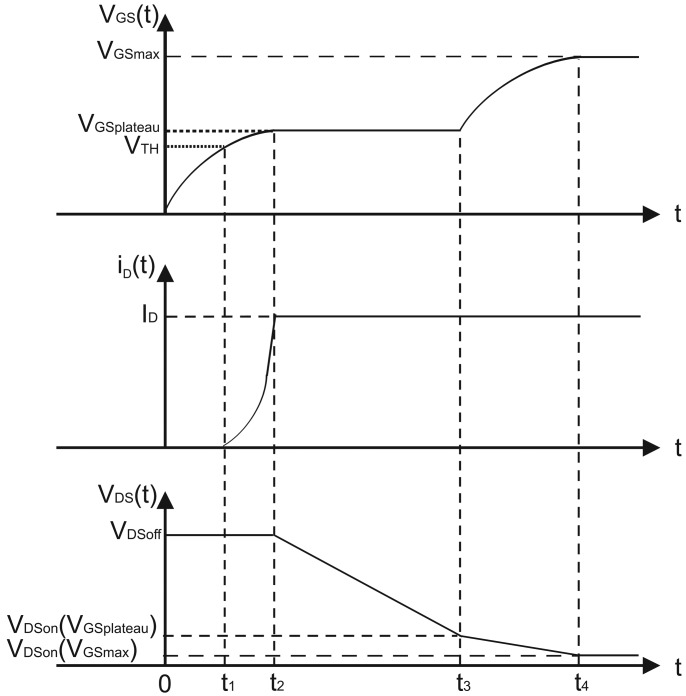
Waveforms for the power MOSFET during turn-on with a gate voltage source.

The switching behavior of Power MOSFET structures is governed by the gate drive circuit and the nature of the load. The power transistor is switched on and off by a control or gate drive circuit, which can be represented (Thevenin's equivalent) as a DC voltage *V*_G_ with a series resistance *R*_G_. The load current *i*_L_ transfers between the power MOSFET device and the freewheeling diode during each operating cycle. The inductor is charged and its current is increased when the Power MOSFET is turned-on while it is discharged when the load current flows through the diode. However, the change in the inductor current is small during one cycle, allowing the assumption that the current *I*_L_ is constant. On the basis of that, a new current sensor that operates during the transient of the Power MOSFET has been conceived. To this purpose, the waveform of the transient of a Power MOSFET is represented in Figure 3 and described in the following. Whenever the Power MOSFET is in off-state, the load current flows through the freewheeling diode. The initial conditions for the Power MOSFET are defined by *v*_GS_(0) = 0, *i*_D_(0) = 0, *v*_DS_(0) = *V*_DS_OFF__. During the turn-on process, the gate bias voltage source *V*_G_ starts to charge the capacitances of the Power MOSFET. Since no drain current can flow through the power MOSFET device until the gate voltage exceeds its threshold voltage, the drain voltage initially remains at the drain bias voltage. The gate–drain capacitance *C*_GD_(*V*_DS_OFF__) remains constant because the drain voltage is constant. Consequently, the time constant for charging the gate of the power MOSFET device is *R*_G_ · [*C*_GS_ + *C*_GD_(*V*_DS_OFF__)], resulting in a gate voltage given by

(2) $$v_{\text{GS}}(t)=V_{{\text{GS}}_{\max}}\cdot \left[1-e^{-\frac{t}{R_{\text{G}}\cdot [C_{\text{GS}}+C_{\text{GD}}(V_{{\text{DS}}_{\text{OFF}}})]}}\right]$$

and represented in Figure 3. The gate voltage reaches the threshold voltage at time *t*_1_:

(3) $${v_{\text{GS}}(t)|}_{\text{t}={\text{t}}_{1}}=V_{\text{TH}}$$

(4) $$t_{1}=R_{\text{G}}\cdot [C_{\text{GS}}+C_{\text{GD}}(V_{{\text{DS}}_{\text{OFF}}})]\cdot \text{ln}\left(\frac{V_{{\text{GS}}_{\max}}}{V_{{\text{GS}}_{\max}}-V_{\text{TH}}}\right)$$

Once the gate voltage exceeds the threshold voltage, drain current begins to flow.

(5) $$i_{\text{D}}(t)=\frac{\mu_{\text{ni}}C_{\text{OX}}W_{\text{CH}}}{L_{\text{CH}}}\cdot {\left[v_{\text{GS}}(t)-V_{\text{TH}}\right]}^{2}$$

Although the drain current increases, the drain voltage remains at the drain supply voltage *V*_DS_OFF__ because the diode cannot sustain any voltage until all of the load current is transferred to the Power MOSFET. Since the drain voltage remains constant, the drain–gate capacitance is also invariant in the range [*t*_1_ – *t*_2_]. Consequently, the gate voltage continues to increase at an exponential rate as described by Equation (2) with the same time constant. The drain current increases as the square of the gate voltage as described by Equation (5) with a nonlinear waveform as represented in Figure 3.

The drain current increases until it becomes equal to the load current *I*_D_ = *I*_L_ and *v*_GS_(*t*) reaches the voltage plateau *V*_GS_plateau__ at the time *t*_2_.

(6) $${v_{\text{GS}}(t)|}_{\text{t}={\text{t}}_{2}}=V_{{\text{GS}}_{\text{plateau}}}=V_{{\text{GS}}_{\max}}\cdot \left[1-e^{-\frac{t_{2}}{R_{\text{G}}\cdot [C_{\text{GS}}+C_{\text{GD}}(V_{{\text{DS}}_{\text{OFF}}})]}}\right]$$

(7) $$t_{2}=R_{\text{G}}\cdot [C_{\text{GS}}+C_{\text{GD}}(V_{{\text{DS}}_{\text{OFF}}})]\cdot \text{ln}\left(\frac{V_{{\text{GS}}_{\max}}}{V_{{\text{GS}}_{\max}}-\sqrt{\frac{I_{\text{D}}L_{\text{CH}}}{\mu_{\text{n}}C_{\text{ox}}W_{\text{CH}}}-V_{\text{TH}}}}\right).$$

All of the load current has transferred from the diode to the Power MOSFET device at time *t*_2_ and the diode is now able to support voltage. The drain-source voltage of the Power MOSFET starts to reduce at this time. Since the drain current is constant and equal to the load current (*I*_D_ = *I*_L_), the gate voltage at time *t*_2_ can be also expressed as

(8) $${v_{\text{GS}}(t)|}_{{\text{t}}_{2}-{\text{t}}_{3}}=V_{{\text{GS}}_{\text{plateau}}}=V_{\text{TH}}+\sqrt{\frac{I_{\text{D}}L_{\text{CH}}}{\mu_{\text{n}}C_{\text{ox}}W_{\text{CH}}}}.$$

The gate-source voltage remains constant at the plateau voltage until the drain-source voltage has reduced to the on-state voltage drop corresponding to the product of the load current and the on-resistance of the device at a gate bias equal to the plateau voltage. Since the gate voltage is constant during the plateau phase, all the gate current *i*_G_plateau__ is used to charge the gate–drain or Miller capacitance. The gate current during the plateau phase is given by

(9) $$i_{{\text{G}}_{\text{plateau}}}=\frac{V_{{\text{GS}}_{\max}}-V_{{\text{GS}}_{\text{plateau}}}}{R_{\text{G}}}.$$

As this current charges the gate–drain capacitance, its voltage decreases at a rate given by

(10) $$\frac{dv_{\text{GD}}(t)}{dt}=-\frac{i_{\text{GP}}(t)}{C_{\text{GD}}(v_{\text{DS}}(t))}.$$

Since the gate–source voltage is constant at *V*_GS_plateau__ during this time, the drain voltage also decreases linearly with time:

(11) $$\frac{dv_{\text{DS}}(t)}{dt}=\frac{dv_{\text{GD}}(t)}{dt}=-\frac{i_{\text{GP}}(t)}{C_{\text{GD}}(v_{\text{DS}}(t))}=-\frac{V_{{\text{GS}}_{\max}}-V_{{\text{GS}}_{\text{plateau}}}}{R_{\text{G}}\cdot C_{\text{GD}}(v_{\text{DS}}(t))}.$$

At the end of the plateau phase at time *t*_3_, the drain–source voltage becomes equal to the on-state voltage drop corresponding to the plateau gate bias voltage. Based on this, the duration of the plateau can be expressed as:

(12) $$\int_{t_{2}}^{t_{3}}dv_{\text{D}}=\int_{t_{2}}^{t_{3}}-\frac{V_{{\text{GS}}_{\max}}-V_{{\text{GS}}_{\text{plateau}}}}{R_{\text{G}}\cdot C_{\text{GD}}(v_{\text{DS}}(t))}dt$$

(13) $$t_{3}-t_{2}=\frac{R_{\text{G}}\cdot C_{{\text{GD}}_{\text{AVG}}}}{V_{{\text{GS}}_{\max}}-V_{{\text{GS}}_{\text{plateau}}}}\cdot [V_{{\text{DS}}_{\text{OFF}}}-I_{\text{D}}R_{{\text{DS}}_{\text{ON}}}(V_{{\text{GS}}_{\text{plateau}}})]$$

where *C*_GD_av__ is the gate–drain capacitance assumed to have a constant average value during the transient and *R*_DS_ON__(*V*_GS_plateau__) is the on-resistance of the power MOSFET device for a gate-source voltage equal to the plateau voltage. Beyond the plateau, the gate voltage increases exponentially again as shown in the Figure 3 until it reaches the gate supply voltage. The time constant for this exponential rise is different from the initial phase due to the large gate–drain capacitance. The increasing gate voltage produces a reduction of the on-resistance of the power MOSFET device, resulting in a small reduction of the drain voltage during this fourth phase of the turn-on process.

Considering reasonable value for the parasitics of the Power transistor sized in the order of *mm*^2^ and for the integrated driving resistance *R*_G_, the interval time (*t*_3_ – *t*_2_) results to be larger than hundreds of nanoseconds. On the basis of a trigger event occurring during this interval, a new current sensor is proposed in Section 4.

## 4. A New Current Sensor Based on the Miller Effect

In order to design a current sensor immune to EMI, a new circuit is conceived, which is not electrically connected with the Power MOSFET drain terminal that is prone to disturbances. To this purpose, the current that flows through the Power MOSFET (*i*_D_) is mirrored and processed during the *V*_GS_ voltage plateau due to the Miller effect (between points 2 and 3 in Figure 3). In this region, the Power MOSFET operates in the saturation region and the mirrored current *I*_MIRROR_ is not dependent on the drain-source voltage and is only related with the mirroring factor K. The Power MOSFET is driven by a PWM signal that provides both the proper-timing driving voltage and a negative pulse that generates a switching transient with negligible reduction of the current in inductive loads.

**Figure 4.**
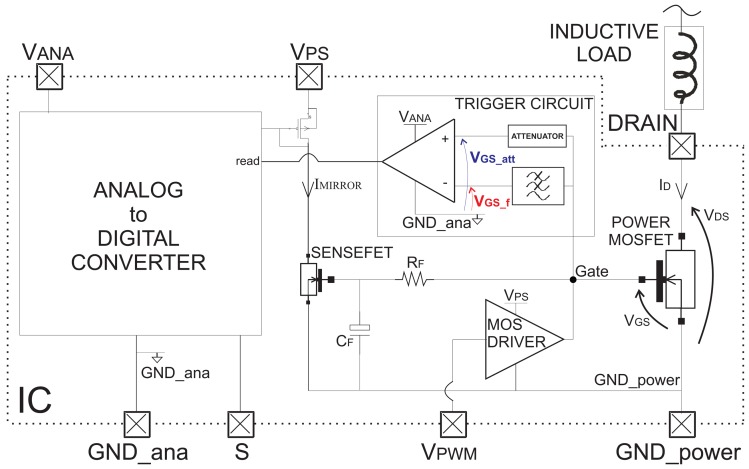
Block diagram of the proposed current sensor that exploits the Miller effect.

**Figure 5.**
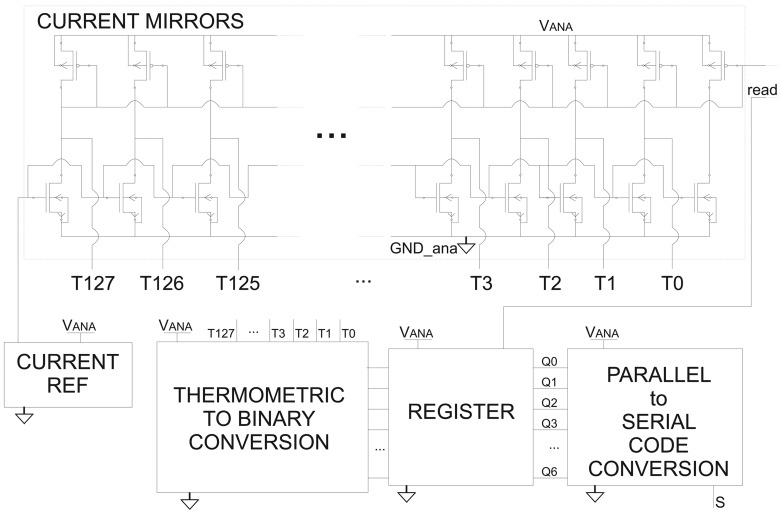
Analog to Digital Converter in Figure 4.

The block diagram of the proposed current sensor is shown in Figure 4. A low-pass RC filter (*R*_F_*– C*_F_) with a cut-off frequency significantly lower than the frequency of the EMI disturbances is placed between the Power MOSFET and SenseFET gates. In particular, *R*_F_ = 10 *k*Ω and *C*_F_ = 2 *pF*, so that the cut-off frequency is less than 10 MHz (*f*_CUT_OFF_ ≈ 8 *MHz*). The current *I*_unknown_ is mirrored by a SenseFET, processed and converted in a serial code (Analog to Digital Converter in Figure 4, further reported in Figure 5). The SenseFET current, in turn, is mirrored 128 times. Each of these currents is compared with progressively scaled current references in order to provide a thermometric digital code of the SenseFET current *I*_MIRROR_. Then, a further thermometric-to-binary conversion is provided [20]. As soon as a trigger event occurs, the binary code is written in a register. Such a binary word provides the value of the current to detect *I*_unknown_, which is then converted to a serial code.

**Figure 6.**
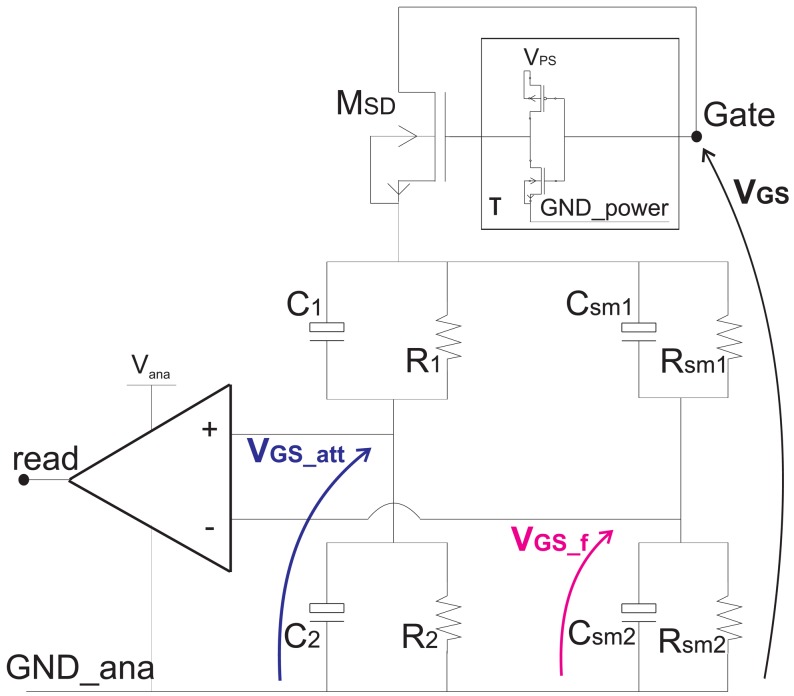
Trigger circuit.

The trigger circuit is based on the comparison of an attenuated gate-source characteristic *V*_GS_att__ with the gate-source voltage after a low-pass filtering process *V*_GS_f_. Both these two voltages are obtained from the gate-source voltage *V*_GS_ by means of only passive component as represented in Figure 6. An inverting stage made of a high voltage inverter allows to null the current dissipation whenever the *V*_GS_ voltage transient ends. Neglecting the drop voltage across the transistor *M*_SD_ that operates in triode during the transient, the voltage V_GS_att_ is given by:

(14) $$V_{\text{GS}\_\text{att}}=\frac{R_{2}(1+sR_{1}C_{1})}{R_{2}(1+sR_{1}C_{1})+R_{1}(1+sR_{2}C_{2})}V_{GS}$$

Setting *R*_1_*C*_1_ = *R*_2_*C*_2_ the voltage *V*_GS_att__ is simply the gate-source voltage *V*_GS_ scaled by a factor *k_din_adp_* independently by the frequency components of the switching transient of the Power MOSFET.

(15) $$V_{\text{GS}\_\text{att}}=\frac{R_{2}}{R_{1}+R_{2}}V_{\text{GS}}=\frac{C_{1}}{C_{1}+C_{2}}V_{\text{GS}}=k_{\text{din}\_\text{adp}}\cdot V_{\text{GS}}$$

where *k_din_adp_* is set in order to not overcome the maximum voltage deliverable to the analog blocks.

(16) $$k_{\text{din}\_\text{adp}}=\frac{R_{2}}{R_{1}+R_{2}}=\frac{C_{1}}{C_{1}+C_{2}}$$

**Figure 7.**
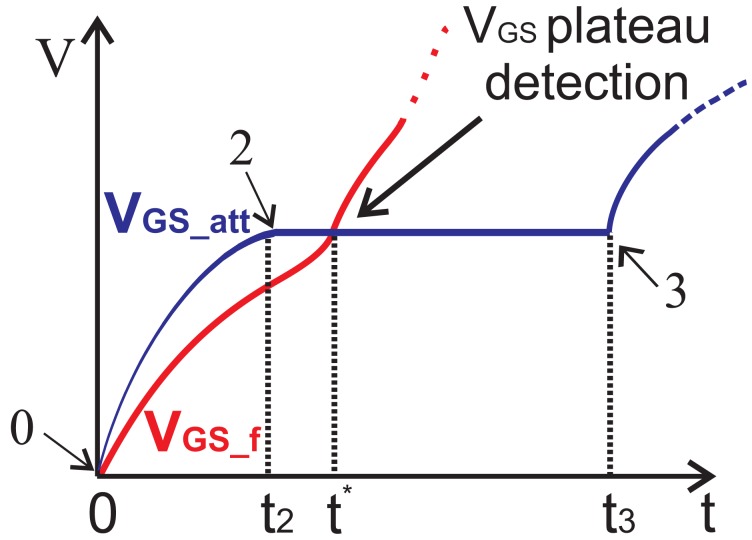
Principle of the detection of the gate-source plateau.

The voltage *V*_GS_f_ is obtained by a filtering and the passive components (*R_sm_*_1_, *C_sm_*_1_, *R_sm_*_2_ and *C_sm_*_2_) are set to makes this voltage *V*_GS_f_ lower than the attenuated voltage *V*_GS_att__ during the interval [0 – *t*_1_]. At the same time, the passive components are set such that the filtered voltage *V*_GS_f_ reaches the attenuated voltage *V*_GS_att__ at the time t* during the plateau, as sketched in Figure 7.

(17) $$\{\begin{array}{ll} V_{\text{GS}\_\text{f}}(t)<V_{\text{GS}\_\text{att}}(t) & \text{if}\;0<t<t*\\ V_{\text{GS}\_\text{f}}(t)>V_{\text{GS}\_\text{att}}(t) & \text{if}\;t*<t<t_{3} \end{array}$$

Assuming *V*_GS_ as a ramp before the voltage plateau, it can be shown that the filtered voltage *V*_GS_f_(*t*) can be expressed as:

(18) $$V_{\text{GS}\_\text{f}}(t)=\{\begin{array}{ll} \frac{mG}{{\left(s_{p}\right)}^{2}}(s_{p}-s_{z})(1-e^{-s_{p}\cdot t})+\frac{mGs_{z}}{s_{p}}\cdot t & \text{if}\;0<t<t_{2}\\ \frac{mG}{s_{p}^{2}}(s_{p}-s_{z})e^{-s_{p}\cdot t}(e^{s_{p}\cdot t_{2}}-1)+\frac{mGs_{z}}{s_{p}}\cdot t_{2} & \text{if}\;t_{2}<t<t_{3} \end{array}$$

where

(19) $$m=\frac{V_{TH}}{t_{1}}$$

(20) $$G=\frac{C_{sm1}}{C_{sm1}+C_{sm2}}$$

(21) $$s_{z}=\frac{1}{R_{sm1}\cdot C_{sm1}}$$

(22) $$s_{p}=\frac{R_{sm1}+R_{sm2}}{R_{sm1}\cdot R_{sm2}\cdot (C_{sm1}+C_{sm2})}$$

On the basis of the two considered voltages *V*_GS_f_ and *V*_GS_att_, it is possible to find out the time t* during the voltage plateau when they are equal.

(23) $${V_{\text{GS}\_\text{f}}(t*)|}_{\left(t_{2}<t*<t_{3}\right)}=k_{\text{din}\_\text{adp}}V_{{\text{GS}}_{\text{plateau}}}$$

(24) $$\frac{mG}{s_{p}^{2}}(s_{p}-s_{z})e^{-s_{p}\cdot t*}(e^{s_{p}\cdot t_{2}}-1)+\frac{mGs_{z}}{s_{p}}\cdot t_{2}=k_{\text{din}\_\text{adp}}V_{GS_{\text{plateau}}}$$

(25) $$t*=-\frac{1}{s_{p}}\cdot \text{ln}\left[\frac{k_{\text{din}\_\text{adp}}V_{GS_{\text{plateau}}}-\frac{mGs_{z}}{s_{p}}t_{2}}{\frac{G}{s_{p}^{2}}(s_{p}-s_{z})(e^{s_{p}\cdot t_{2}}-1)}\right].$$

Firstly, a minimum value for *C_sm_*_1_ = 200 fF is set and a reasonable value of *R_sm_*_1_ = 62 *k*Ω is chosen to set the zero *s_z_* = 12.8 *MHz*. Consequently, the value of *C_sm_*_2_ = 2.5 *pF* and *R_sm_*_2_ = 19 *k*Ω are set in order to place the pole *s_p_* = 4.05 *MHz* before the zero. In this way an instant t* in the range [ *t*_2_ – *t*_3_] according to Equation (25) is found out and a correct trigger event can be provided. As soon as the mentioned two voltages *V*_GS_att_ and *V*_GS_f_ reach the same value during the gate-source plateau voltage due to the Miller effect, a comparator finds out a trigger event. Such an event enables to write the register whenever the Power MOSFET is in saturation during its switching transient. In the register, the digital value of the SenseFET current *I*_MIRROR_ is stored. Such a value represents the current to sense *I*_unknown_ scaled by the mirroring ratio K.

The proposed circuit is suitable for inductor current monitoring in DC-DC converter that operates in Continuous Conduction Mode (CCM) and whenever the detection of the current flowing in a Power MOSFETs operating in PWM that drive resistive and inductive loads is needed. The current to be monitored ranges from 100 *μA* to 5 A, and the mirroring factor K has been set **K** = 10^4^. The sensibility of the designed sensor is 50 *μA* (LSB). The absence of the amplifier provides an higher immunity to EMI and a faster response in inductor-current monitoring than the traditional current-sensing technique as shown in Section 5.

## 5. Prediction of Integrated Current Sensors to RFI

This section shows the results of the time-domain simulations carried out to evaluate the susceptibility of the proposed current sensor and the traditional sensor to RFI.

The proposed new current sensor is based on the transient behavior of the Power MOSFET in which the current to detect flows, and such a transient strongly depends on the parasitics of the Power transistor itself. For this reason, no other additional parasitic capacitances due to the test circuit have to affect the transient behavior of the Power MOSFET. Therefore, a CW RFI current of magnitude *i*_RF_ = 200 mA has been superimposed onto the current to detect *I*_D_ by means of a toroidal RF transformer.

**Figure 8.**
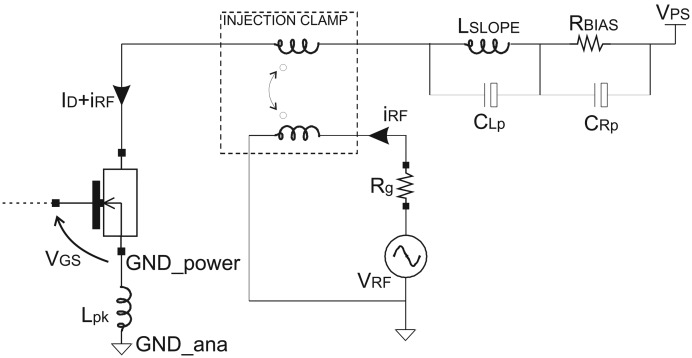
Schematic view of the Power MOSFET considered in the time-domain simulations.

In fact, the analyses of the susceptibility to RFI have been carried out referring to the schematic view of Figure 8 similarly to immunity tests [21]. A resistor *R*_BIAS_ sets the value of the current *I*_D_.

**Figure 9.**
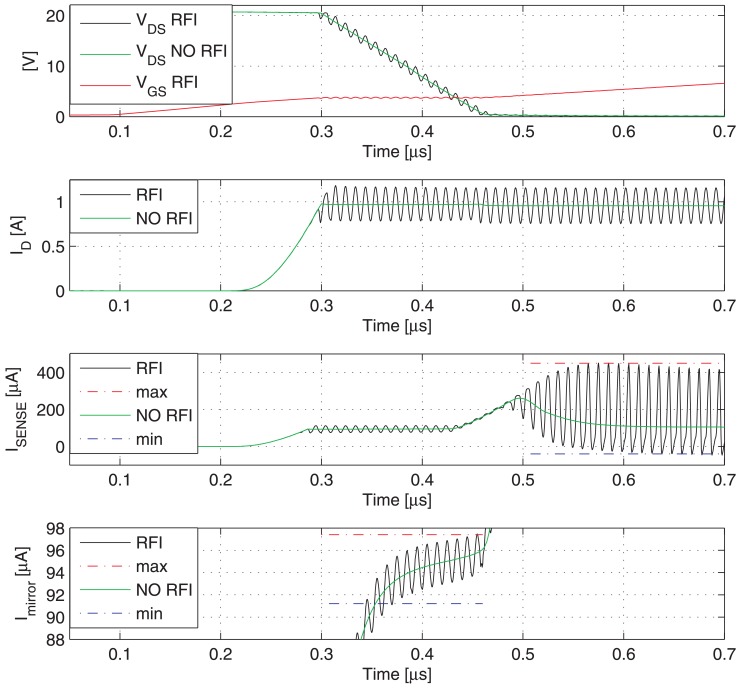
Switching transient traces of a Power MOSFET (*V*_DS_, *V*_GS_, *I*_D_) and the respective currents provided by a traditional SenseFET sensor (*I*_SENSE_) and the new sensor based on the Miller effect (*I*_MIRROR_). CW RFI current amplitude 200 mA @ 100 MHz superimposed on the drain current.

**Figure 10.**
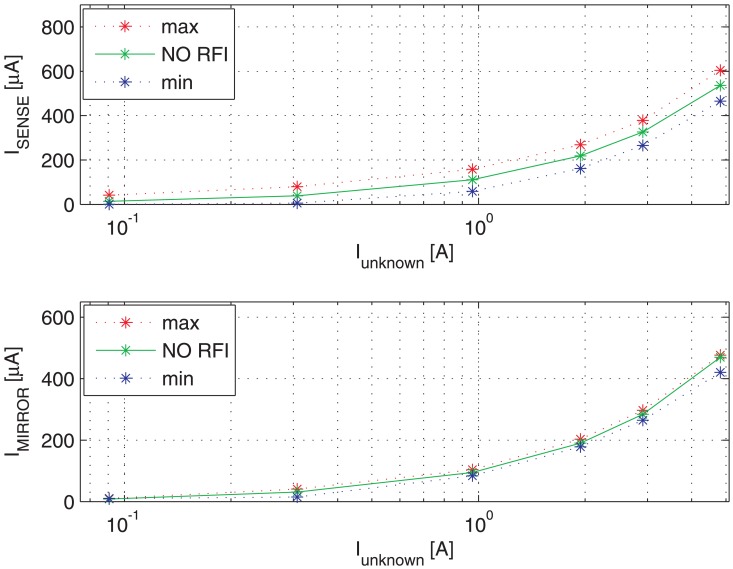
Maximum and minimum current-sensing of the traditional SenseFET current sensor (*I*_SENSE_) and the new sensor based on the Miller effect (*I*_MIRROR_) *versus* current to sense *I*_D_. CW RFI current amplitude 200 mA @ 10 MHz.

**Figure 11.**
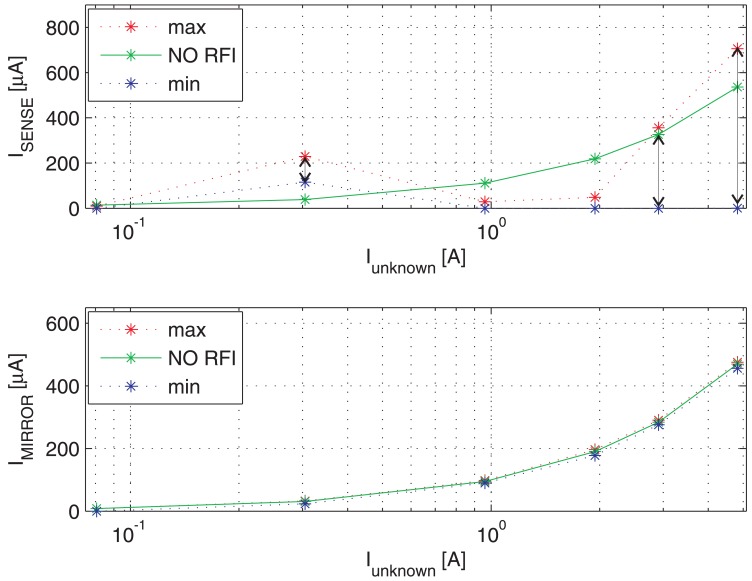
Maximum and minimum current-sensing of the traditional SenseFET current sensor (*I*_SENSE_) and the new sensor based on the Miller effect (*I*_MIRROR_) *versus* current to sense *I*_D_. CW RFI current amplitude 200 mA @ 100 MHz.

**Figure 12.**
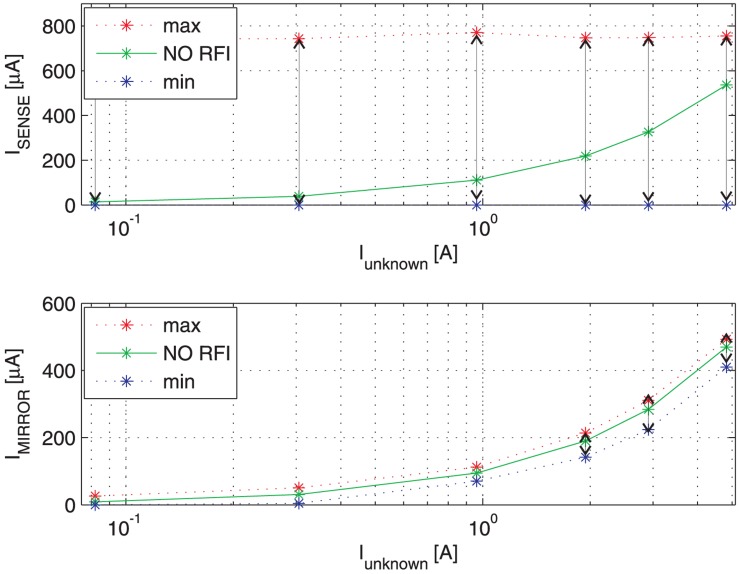
Maximum and minimum current-sensing of the traditional SenseFET current sensor (*I*_SENSE_) and the new sensor based on the Miller effect (*I*_MIRROR_) *versus* current to sense *I*_D_. CW RFI current amplitude 200 mA @ 400 MHz.

**Figure 13.**
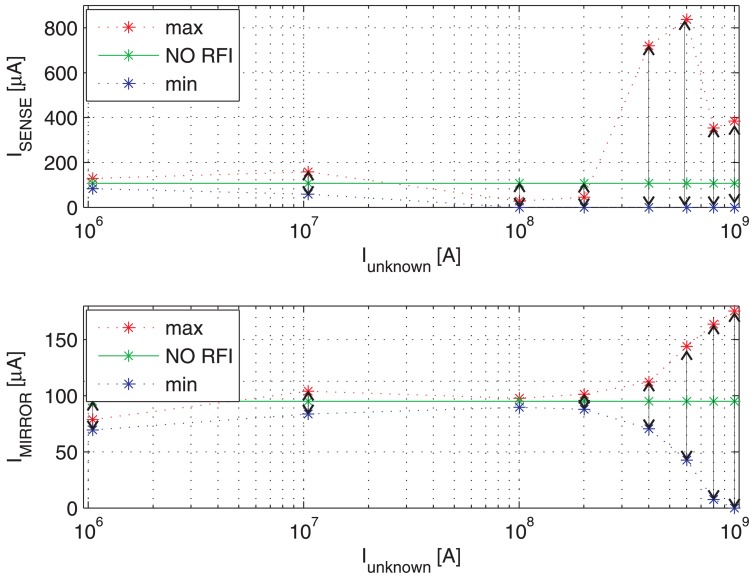
Maximum and minimum current-sensing of the traditional SenseFET current sensor (*I*_SENSE_) and the new sensor based on the Miller effect (*I*_MIRROR_) *versus* frequency. CW RFI current amplitude 200 mA superimposed on a current *I*_D_ = 1A.

Figure 9 shows the switching transient traces (*V*_DS_, *V*_GS_) of a Power MOSFET in which the current to be detected *I*_D_ flows, as well as the respective currents provided by a traditional SenseFET sensor (*I*_SENSE_) and the proposed new sensor (*I*_MIRROR_). In such a time-domain simulations, a CW RFI current *i*_RF_ = 200 *mA* at 100 MHz superimposed on the drain current have been considered. The currents provided by the traditional SenseFET sensor (*I*_SENSE_) and by the proposed new sensor (*I*_MIRROR_) versus current to sense *I*_D_ and their minimum and maximum errors due to the aforementioned RFI magnitude at 10 MHz, 100 MHz and 400 MHz are shown respectively in Figures 10–12. Furthermore, the minimum and maximum errors of the two considered currents due to a CW RFI *i*_RF_ = 200 *mA* superimposed on the drain current *I*_D_ versus frequency are reported in Figure 13. The results highlight that the immunity of the proposed current sensor to RFI is significantly greater than that of the conventional SenseFET circuit shown in Figure 1.

## 6. Conclusions

In this work, the susceptibility of common integrated sensors to RFI has been discussed for power transistor current monitoring. In order to improve the immunity of Power MOSFET transistor to RFI in current sensing, a new integrated solution has been proposed. It exploits the Miller effect on the switching transient of the Power MOSFET. The detection of the gate-source voltage plateau due to the Miller effect allows to mirror the current to be detected whenever the Power MOSFET operates in saturation. In this way, the mirrored current is not dependent on the drain-source voltage and the RFI that reaches the drain terminal of the Power MOSFET does not strongly influence the operation of the current sensor. Furthermore, there is no need to connect any amplifier to the drain terminal of the Power MOSFET, hence eliminating a barrier on the overall current sensor performance. The operation of the new current sensing circuit and that of a conventional current sensor have been compared in time-domain simulations. Since the novel sensor operates during the switching transient, it provides a faster current detection than the traditional sensor. Whereas the amplifier usually limits the performance of the SenseFET-technique, no such issue has to be considered for the new sensor. The susceptibility of the proposed current monitoring solution has been evaluated through time-domain simulations and compared with that of a conventional current sensor. The analyses carried out in this work have shown a significant improvement in the immunity to RFI in the range 1 MHz–1 GHz that the new proposed solution enables.
